# Examining the “Night Break” Method in *Cannabis sativa* Horticulture: Vegetative Daily Light Integral Affects Yield of Extractable Biomass in *C. sativa*

**DOI:** 10.3390/plants14193095

**Published:** 2025-10-08

**Authors:** Evan F. Grover, Samuel R. Haiden, Gerald A. Berkowitz

**Affiliations:** Agricultural Biotechnology Laboratory, Department of Plant Science and Landscape Architecture, University of Connecticut, Storrs, CT 06269, USA

**Keywords:** night break, daily light integral, photosynthetic photon flux density, *Cannabis sativa*

## Abstract

*Cannabis sativa* is a short-day (SD) plant, producing inflorescences when the daily scotoperiod (period of darkness) exceeds approximately 10 h of length. As such, the vegetative photoperiod is typically maintained at 16 to 18 h, which limits the scotoperiod to no more than 6 to 8 h and keeps plants in the vegetative stage. The electricity cost associated with supplemental lighting is a major concern for controlled environment cannabis cultivation. Therefore, the strategy of utilizing a 12 h photoperiod while interrupting the scotoperiod with a 1 h “night break” (NB) is appealing, as it reduces the overall electricity required for supplemental lighting by nearly one third, while maintaining vegetative growth. Our study tested the feasibility of this method under controlled indoor conditions. We studied the effect of the NB method (as compared to conventional light/dark periods) on cannabinoid and extractable biomass yields, as well as phenotype. Reducing vegetative DLI via the NB method (29.4 → 21.2 mol m^−2^ d^−1^) reduced extractable floral biomass by ~22% (control 1295 g vs. NB 1015 g per tent), while cannabinoid concentrations were similar between treatments. We also found that NB plants were less vigorous and shorter, with shorter internodes and fewer branches. This evidence suggests that although the NB method may reduce electricity costs during the vegetative stage of controlled environment cannabis growth, the method is not economically feasible due to the loss of yield and plant vigor.

## 1. Introduction

*Cannabis sativa* is a photoperiod-sensitive flowering annual short-day (SD) plant. As a crop, it is gathering increased significance both economically and industrially for its medicinal oils, edible oil, seeds, fiber, and capacity for phytoremediation [1,2]. Across all latitudes, cannabis cultivators must concern themselves with extending the photoperiod to maintain vegetative growth long enough to produce healthy, vigorous plants that will produce optimal flower and cannabinoid yields upon initiation of the reproductive phase of growth. It has been shown in many species, as well as in *C. sativa*, that the activation of a FLOWERING LOCUS T (FT)-like transcription factor initiates the meristematic transition to flowering [3,4,5]. Expression of *FT* is activated by a network of genes whose expression is entrained and regulated by circadian rhythms [6,7,8]. Photoreceptors such as phytochromes, cryptochromes, and phototropins perceive changes in light quality, duration, and direction, and interact to modulate photoperiodic flowering responses [9,10,11]. Flower induction is governed by photoreceptors and circadian networks that measure night length. When the scotoperiod exceeds the cultivar specific critical duration, often near 10 to 11 h, signaling through PHYTOCHROME A (PHYA), PHYTOCHROME B (PHYB), CRYPTOCHROME (CRY), PHYTOCHROME INTERACTING FACTORS (PIFs), EARLY FLOWERING 3 (ELF3), GIGANTEA (GI), CONSTANS (CO), and PSEUDO RESPONSE REGULATOR 37 (PRR37) shifts to favor expression of *FT*, triggering reproductive development [5,12]. However, the entrainment of these photoreceptors and circadian-clock proteins is determined not by the photoperiod but by the scotoperiod. By interrupting the scotoperiod, regardless of the overall photoperiod duration, flowering can be suppressed [13]. Daylength extension (DE) is a commonly used lighting strategy where artificial light is added at the end of the natural daylight period **to** prolong the total photoperiod and prevent flowering in short-day plants, though recent research has shown that an interruption of the dark period is equally effective in maintaining vegetative growth [14]. This technique is commonly used in greenhouses with supplemental lighting, or in controlled environment systems with no natural sunlight where artificial light is required, and is referred to as the “night break” (NB) method.

The night break (NB) method, also referred to as night interruption, involves providing a short period of light during the night and is commonly used to control flowering in short-day plants like many flowers and vegetables. This technique effectively extends the perceived day length, delaying flowering and promoting vegetative growth, which allows growers to better manage crop timing and yield. Kim et al. [15] showed that night interruption with specific light intensities increased both vegetative growth and flowering rates in *Cymbidium* orchids, demonstrating how NB can be applied to improve commercial horticulture production. Similarly, in *Chrysanthemum*, irregular night break lighting increased carbon gain, which was strongly correlated with both daylength and daily light integral (DLI). This response occurred regardless of the irregularity of the light breaks and despite disruptions to circadian-regulated processes such as carbohydrate metabolism, chlorophyll fluorescence, and chlorophyll content, while still promoting leaf expansion and stem elongation [16]. DLI is a quantification of the cumulative photosynthetic photon flux density (PPFD) plants receive daily and plays a critical role in influencing photosynthate production during the vegetative stage of *C. sativa* [17]. High DLI exposure during this stage promotes robust vegetative growth by enhancing photosynthetic rates, leading to increased biomass accumulation that serves as a structural and metabolic foundation for future flower production [18]. Research has demonstrated that optimized light during vegetative growth not only supports the development of extensive root and shoot systems but also influences the plant’s overall capacity to produce flowers under controlled photoperiod conditions [19], revealing that floral initiation was most successful under 12.0- and 13.2 h photoperiods, corresponding to uninterrupted dark periods of 12 and 10.8 h, respectively [20]. This underscores the importance of precise light management strategies, such as adjusting DLI through controlled light cycles, to maximize both vegetative performance and subsequent flowering potential in cannabis cultivation systems [21]. By using a 12 h photoperiod followed by a 1 h NB to break up the dark cycle, growers can ensure that plants continue vegetative growth without needing a full 18 h photoperiod. This strategic interruption of the scotoperiod with an hour of light can be used to artificially extend the vegetative phase, allowing plants more time to develop leaves and stems crucial for supporting future flower growth. While it is possible to compensate for the loss of DLI resulting from a 13 h photoperiod (compared to 18 h) by increasing the intensity of the photosynthetically active radiation (PAR), it has been shown that photosynthesis and water use efficiency in *C. sativa* increases with light intensity peaking at 1500 μmol m^−2^s^−1^ PPFD before declining at higher light levels [22]. Flower yields, however, have been shown to increase with higher light intensity at the canopy level, with gains continuing up to 1800 μmol m^−2^·s^−1^ [17]. Transpiration and stomatal conductance was found to rise with increasing light and temperature across all tested conditions [17,22]. If the grower compensated for the shortened 13 h photoperiod in the NB group by increasing the PPFD by approximately 30%, this would only be effective if the total resulting PAR intensity remained below 1500 PPFD.

Furthermore, this would defeat the purpose of saving energy. By increasing energy inputs to increase PAR to compensate for lost DLI, the grower will ultimately increase their energy expenditure, and the method would not be advantageous on an economic basis. As such, we decided not to compensate for lost DLI by increasing PAR, as this would defeat the purpose of reducing energy usage. To our knowledge, no peer-reviewed studies have directly investigated the use of the night break (NB) method in *C. sativa* as a strategy to reduce vegetative DLI and electricity consumption while assessing impacts on yield and secondary metabolite production. This study aims to fill that gap by evaluating whether reduced vegetative DLI via NB lighting compromises plant vigor, biomass accumulation, or cannabinoid content. We hypothesized that implementing the NB method to reduce vegetative DLI would result in a modest reduction in floral biomass, but that cannabinoid yield and composition would remain largely unaffected. We further anticipated that the reduction in electricity usage from the shortened photoperiod would be sufficient to offset any yield losses, thereby rendering the NB strategy economically viable for controlled environment cannabis production.

## 2. Methods

### 2.1. Vegetative Phase

A total of 16 *C. sativa* (Wife) clones were taken from a stock mother plant, separated into two groups of 8, and grown vegetatively in two 5 × 5 Gorilla Grow Tents (Gorilla Grow Tent, Inc., Santa Rosa, CA, USA) for this study. Cuttings were rooted using Clonex^®^ Rooting Gel (Growth Technology Ltd., Taunton, Somerset, UK) for 21 days before the most uniform clones were potted in #900 blow molded nursery pots in Promix-BX (Premier Tech Horticulture, Rivière-du-Loup, QC, Canada) supplemented with Osmocote^®^ (ICL Specialty Fertilizers, Summerville, SC, USA). Before treatment assignment, clones were screened for uniformity in height, node number, and vigor. Plants were paired by closest match, each pair split between treatments, and placed at identical relative positions to form spatially paired replicates, minimizing early transplant effects. Lighting was provided by Adaptiiv GSTS 1000 full-spectrum LED fixtures (Adaptiiv Grow Technologies, Ridgefield, CT, USA), which emit a broad white spectrum spanning 400–700 nm. The mean canopy-level light intensity was approximately 450 μmol m^−2^ s^−1^ photosynthetic photon flux density (PPFD), measured using an Apogee PS-100 spectroradiometer (Apogee Instruments, Inc., Logan, UT, USA). Jack’s 15-30-15 fertilizer (JR Peters Inc., Allentown, PA, USA) delivered as a constant liquid feed via drip fertigation. Fertigation volume remained consistent with all plants receiving equal time on the same drip irrigation system. For the control tent, the lighting was set to 18 h on and 6 h off. The NB photoperiod consisted of 12 h of light, a 5 h dark interval, a 1 h light pulse during the seventeenth hour of the day, and a final 6 h dark interval. Temperature and relative humidity were equivalent and remained constant between treatment groups. Early measurements showed uniform PPFD across positions; as the canopy developed, the corner plants became slightly less exposed than the center. To address this, before flowering we harvested the four corner plants (plants 1, 3, 7, and 8 from each group) from each tent for vegetative biomass analysis.

### 2.2. Flowering Phase

Reproductive measurements including flower weight and cannabinoid content were taken from the four central plants in each tent that received more uniform light. After harvesting the peripheral plants, the remaining four plants from each treatment were transplanted into #1200 blow molded nursery pots and moved to a greenhouse bay which used an automated blackout curtain system that closed fully during dark periods and opened during the 12 h light interval. When open, plants received natural daylight supplemented by 1000 watt high pressure sodium fixtures; when closed, no external light entered. The curtain fixed the 12 h light and 12 h dark photoperiod, and flowering conditions were identical for both treatments, with DLI matched between groups, so differences in final floral mass reflect vegetative light history. Fertigation and measurements continued as previously.

### 2.3. Measurement of PPFD and Calculation of DLI

PPFD was measured using a calibrated Apogee PS-100 spectroradiometer (Apogee Instruments, Inc., Logan, UT, USA). For each tent, five measurements were taken: one measurement at each of the four corners and a central measurement. This measurement included spectral information and PPFD. A mean of the five measurements was used to determine the average PPFD per tent. Micromoles of light per meter squared per second were multiplied by the photoperiod to yield DLI calculations. Vegetative canopy-level PPFD averaged ~450 μmol m^−2^ s^−1^ during both the main light period and the night-break pulse. DLI was calculated as 29.4 mol m^−2^ d^−1^ for control and 21.2 mol m^−2^ d^−1^ for the night-break treatment. This same process was repeated upon transition to the greenhouse under high pressure sodium (HPS) lighting conditions, with ten measurements rather than five. The mean DLI during the flowering stage was 19.75 mol m^−2^ d^−1^ for control and 19.91 mol m^−2^ d^−1^ for NB, indicating closely matched light exposure.

### 2.4. Phenotypic Analysis

Internodal length, plant height and branch length were measured using a tape measure, ruler, or meter stick. Height measurements were taken weekly by measuring from the crown (base of stem) to the apical bud. Leaf surface area was measured using the LeafScan app (version 1.3.21) [23] using the first three true leaves beneath the apical bud. Roots were separated by washing away potting medium, leaf stem and flower tissues were separated by hand. All biomass samples were weighed using an analytical scale.

### 2.5. Chemotypic Analysis

Immediately after harvest, whole plants were cut and hung to dry in tents for two weeks, with plants from both treatments distributed across tents. After drying, floral tissue was separated from stems and leaves, and the extractable biomass from each plant was milled to homogeneity to create a single plant composite. Cannabinoids were quantified by HPLC using an LC-2050C i-Series system, Cannabis/Hemp Analyzer High Sensitivity configuration (Shimadzu Corporation, Kyoto, Japan; Shimadzu Scientific Instruments, Columbia, MD, USA) with UV-Vis detection at 220 nm and LabSolutions DB control (version 5.111). Separation was performed on a Gemini C18 column, 5 μm, 110 Å, 150 × 2.0 mm (Phenomenex Inc., Torrance, CA, USA). Standard calibration curves were made with a serial dilution of an 11-cannabinoid standard from Shimadzu. 100 mg samples were collected from the milled composite of extractable biomass from a single plant, and extracted into 10 mL of methanol before filtration, dilution and injection. The method was executed according to manufacturer’s directions without modification.

### 2.6. Statistical Analysis

All statistical analyses were performed in Microsoft Excel for Microsoft 365 (version 2408; Microsoft Corporation, Redmond, WA, USA). Differences between control and experimental groups for phenotypic and chemotypic variables were evaluated using a two-tailed, paired Student’s *t*-test. A Student’s *t*-test is mathematically equivalent to a one-way analysis of variance (ANOVA) when only two groups are compared; however, the *t*-test was selected here for its direct applicability and clarity in the case of two group comparisons. Significance levels were denoted as follows: *p* < 0.05 (*), *p* < 0.01 (**), and *p* < 0.001 (***). Data are reported as mean ± standard error of the mean (SEM).

To reduce spatial confounding, plants were assigned as spatial pairs: each control plant was matched to a night break plant occupying the same relative position within its respective tent, and positions remained fixed during the vegetative and flowering growth phases. This position matching was used to minimize microenvironmental variation, while statistical comparisons treated plants as independent observations. Potential outliers were identified on clear technical grounds, such as growth abnormalities not attributable to treatment. When an outlier was removed from one treatment, its spatially paired counterpart was also removed from that analysis to preserve balance. After any such exclusions, sufficient biological replication remained for all tests, with annotation in the figure legends.

## 3. Results

To evaluate the effectiveness of the night break (NB) method in maintaining vegetative growth while reducing energy consumption, we compared morphological, physiological, and chemical characteristics of *C. sativa* plants subjected to two distinct photoperiod treatments. Phenotypic parameters such as plant height, branch number, internodal length, and leaf surface area were tracked weekly to assess vegetative and early flowering development. Biomass measurements were conducted separately for vegetative and reproductive tissues to determine whether reductions in DLI during the vegetative stage affected total growth and yield. Additionally, cannabinoid profiles were analyzed to investigate whether the different light cycles influenced secondary metabolite production. Figures and tables are organized to reflect changes over time and by tissue type, providing a comprehensive overview of plant performance under NB and control conditions. Results were reproducible in a second run performed under similar conditions.

### 3.1. Morphological Development

**Figure 1:**
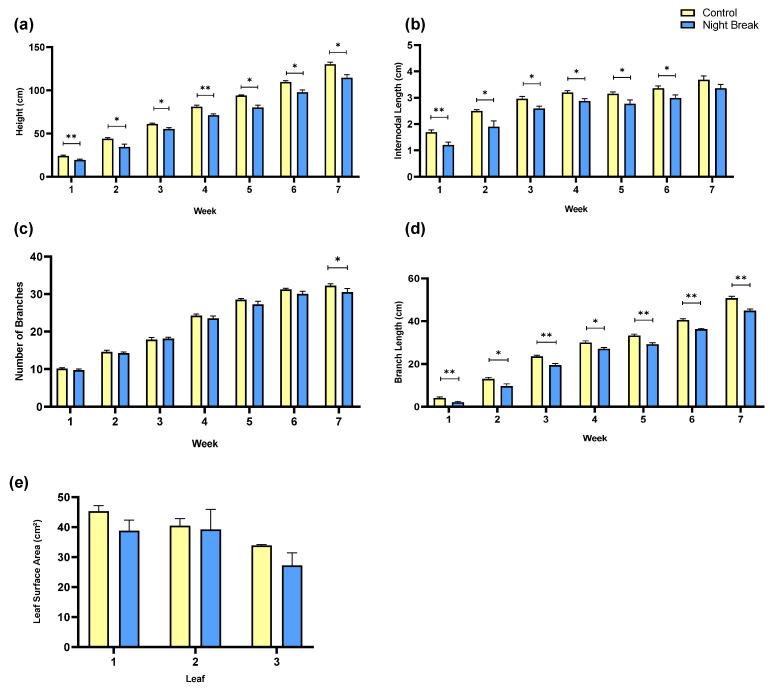
Morphological growth by treatment. (**a**) Weekly plant height means of *C. sativa* by treatment, comparing the average plant height between the control group and the NB group over seven weeks of vegetative (week 1–4) and flowering (week 5–7) growth. (**b**) Weekly means of internodal lengths by treatment. This graph compares the average internodal lengths, or distance between branches on the main stem between the control and the NB group. (**c**) Weekly mean number of branches of *C. sativa* by treatment. This graph compares the number of branches between the control group and the NB group. A statistically significant difference was observed between the treatment groups for week 7. (**d**) Weekly means of axillary branch length by treatment. For each plant we measured every axillary branch from its node of origin on the main stem to the branch tip, then averaged across branches to a plant value and to a group mean. (**e**) Mean leaf surface area of *C. sativa* by treatment. This graph compares the average leaf surface area between the control group and the NB group including the first three true leaves beneath the apical bud. Error bars represent standard error. Asterisks indicate statistically significant differences between treatments at each time point (*p* < 0.05 (*), *p* < 0.01 (**)), determined using an analysis of variance (Student’s paired *t*-test). Similar statistical analyses are shown for Figure 2 and Figure 3.

Differences in morphological development were evident early in the experiment and persisted throughout plant growth and development. Control plants exhibited significantly greater vertical height than NB plants beginning in Week 1 and continuing through Week 7 (Figure 1a). By the end of the experiment, control plants reached an average height of approximately 135 cm, while NB plants averaged just above 110 cm; the NB treatment was associated with a ~19% reduction in this specific measurement of plant growth. This persistent height gap indicates that the reduced length of the NB group’s daily light period negatively impacted plant growth.

The distance between nodes also differed significantly between treatments during the vegetative phase (Figure 1b). Control plants had greater internode lengths from Week 1 through Week 6 (*p* < 0.05 to *p* < 0.001), with week 7 showing no statistical significance but remaining consistent. These findings collectively suggest that the uninterrupted light period promoted more expansive vertical and lateral growth, while the NB treatment induced a more compact plant structure, likely due to reduced total light exposure and cumulative DLI.

In contrast to height and internodal length, treatment effects on the total number of branches were not significantly different until week 7 (Figure 1c). Both the control and NB groups exhibited a steady increase in branching over time, ultimately reaching approximately 30 branches per plant by Week 7. The similar final branch counts between treatments indicate that branch initiation is less sensitive to reduced DLI than elongation growth. Although no statistically significant differences were observed between treatments until the final weekly measurement, the branching trajectories displayed a trend consistent with other growth parameters, where the control group generally maintained slightly higher values than NB plants across the measurement period. When all the branch number measurements for each treatment were pooled over the entire period of data collection (i.e., 7 weeks), the slight reduction in branch number in the night break treatment as compared to the control (~15%) was statistically different (*p* < 0.01).

Similar trends to plant height were observed in lateral growth, as control plants consistently developed longer branches than NB plants (Figure 1d). While both groups experienced steady increases in branch length over time, control plants maintained a statistically significant advantage each week, particularly from Weeks 3–7 (*p* < 0.01 to *p* < 0.001). By harvest, control branches averaged over 50 cm in length compared to approximately 44 cm (a ~12% reduction) in the NB group, suggesting that extended vegetative light exposure supported both vertical and lateral canopy expansion. Despite clear differences in height, branch length, and internode spacing, leaf surface area remained relatively consistent. Although, like the branch number results shown in Figure 1c, the leaf area of plants subjected to the NB treatment were slightly lower (Figure 1e). Measurements of the first three fully expanded leaves below the apical bud revealed no significant differences between groups, though control plants showed a slightly greater surface area for each leaf. Statistical significance may have been found if a greater number of biological replicates were used.

### 3.2. Vegetative and Floral Tissue Biomass

**Figure 2:**
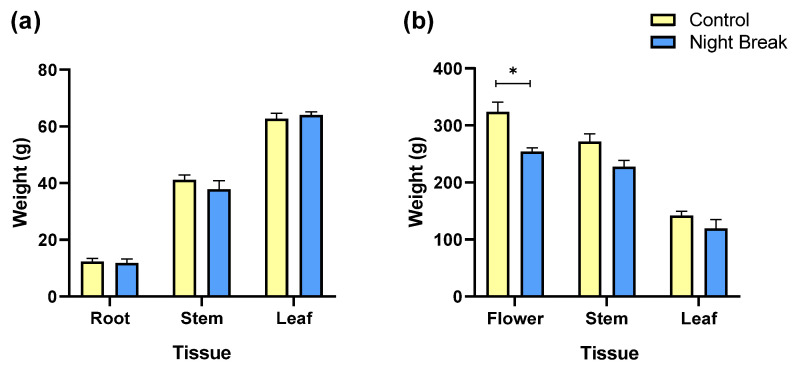
Weights of various tissues measured during the vegetative stage and floral stage. (**a**) This graph compares the mean weight in grams of different plant tissues (root, stem, and leaf). No statistically significant differences were observed between treatments for any tissue type. (**b**) Mean weights of various plant tissues measured during the flowering period by treatment. This graph compares the average biomass of flower, stem, and leaf tissues between the control group and the NB group during the flowering stage.

Biomass allocation followed similar patterns to morphological growth. During the vegetative phase, control plants produced slightly higher stem mass and equivalent leaf and root mass compared to NB plants, though these differences were not statistically significant (Figure 2a). This lack of statistical separation indicates that, despite reduced vertical and branch growth in the NB group, vegetative tissue partitioning remained proportionally similar between treatments. This suggests that plants under NB lighting maintained a comparable balance between root and shoot allocation, even with lower cumulative light exposure.

During the flowering phase, more pronounced differences in biomass distribution emerged (Figure 2b). Flower weight was significantly higher in the control group (*p* < 0.05), with average values exceeding 320 g per plant compared to approximately 250 g in NB plants. Stem mass was also higher in control plants, though not statistically significant, while leaf mass remained similar between treatments. These differences align with the morphological observations from the vegetative stage, where control plants developed slightly longer branches capable of supporting a larger floral load. The extended length of the daily photoperiod during the vegetative growth stage in the control treatment therefore resulted in more robust structural growth, which in turn supported greater floral development during the reproductive phase.

### 3.3. Cannabinoid Composition and Yield

**Figure 3:**
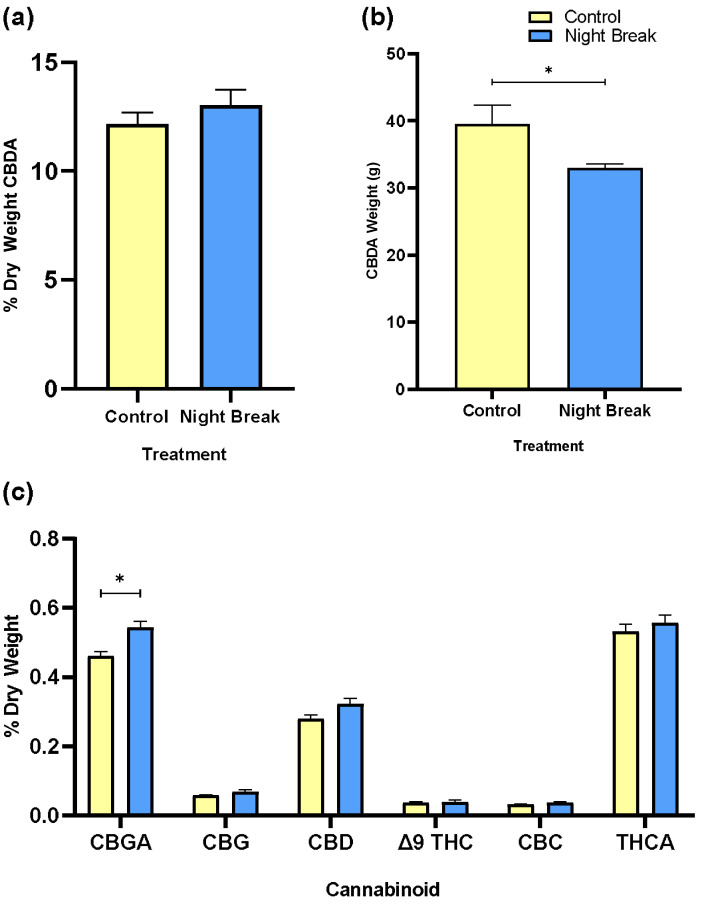
Cannabinoid concentration and yield by treatment. (**a**) Mean percentage of CBDA by dry weight by treatment. This bar chart compares the average percentage of CBDA by dry weight between control and NB treatment groups. (**b**) Mean CBDA weight per plant. This bar chart displays the mean weight of CBDA (in grams) per plant extracted from *C. sativa* plants under their respective light treatments. (**c**) Composite average percentage of cannabinoids by dry weight. This bar chart compares the percentage by dry weight of various cannabinoids including CBGA, CBG, CBD, Δ^9^-THC, CBC, and THCA between control and NB treatment groups.

Cannabinoid concentrations were statistically comparable between the control and NB groups, whereas yield differed. When expressed as a percent of dry weight, CBDA concentration was slightly higher in NB plants than in controls, though not statistically significant (Figure 3a). The total CBDA produced per plant was significantly greater in the control treatment, consistent with the higher flower weight measured for that group.

While the total CBDA yield per plant was higher in the control group (Figure 3b), this outcome primarily reflected the greater floral biomass observed in that treatment. Higher flower production provided more plant material for extraction, resulting in a greater total CBDA yield by weight. Thus, while NB plants showed a small trend toward higher CBDA concentration on a tissue basis, this trend did not overcome the lower floral mass (Figure 3b; see biomass in Table 1), and total CBDA output per plant remained lower than the control. A detailed cannabinoid profile showed that most compounds did not differ significantly between treatments (Figure 3c). However, CBGA was significantly higher in control plants than in NB plants under the paired *t* test (*p* < 0.05), a compound associated with pathway activity during floral development, and a branch point precursor to major cannabinoids via its acidic form [24]. Since the Wife cultivar was selected for high CBDA with typical values ranging from 10–20 percent of flower dry weight, CBDA is shown in its own graph. CBDA concentration in flowers was comparable to levels typical of commercial cannabis despite the relatively low PAR used in this experiment.

### 3.4. Floral and Vegetative DLI

**Figure 4:**
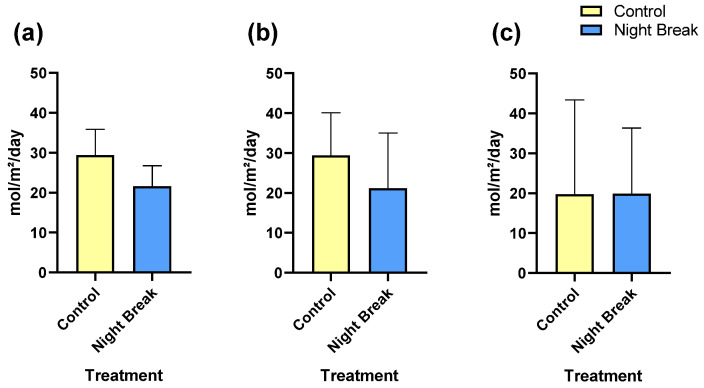
Mean DLI calculated from measurements taken during the beginning and end of the vegetative stage in *C. sativa* plants. (**a**) Mean DLI calculated from initial vegetative light measurements. This bar chart compares the average DLI between control and NB treatment groups at the beginning of the vegetative stage (when rooted clones were potted and placed in tents for treatment). (**b**) Mean DLI at the end of the vegetative stage in *C. sativa* plants. Similarly, this graph compares the average DLI between control and NB treatment groups at the end of the vegetative stage. Both charts exhibit the different DLIs where the NB treatment received less daily cumulative light than the control. (**c**) Mean of DLI measurements taken during the flowering stage in *C. sativa* plants. This bar chart displays the average DLI, measured in moles of light per square meter per day (mol m^−2^ day^−1^), for control and NB treatment groups during the flowering stage. Measurements indicate no meaningful difference in light exposure between the treatment groups during this critical growth phase.

As expected, the NB group received a lower cumulative light dose during the vegetative phase. Average DLI at the start of vegetative growth was approximately 29.4 mol m^−2^ day^−1^ in the control group and 21.2 mol m^−2^ day^−1^ in the NB group, with similar differences observed at the end of the stage (Figure 4a,b). These values confirm that the NB treatment effectively reduced total daily light exposure by shortening the primary photoperiod while inserting a one-hour night interruption to maintain vegetative growth.

During the flowering stage, both groups received comparable DLIs of about 19.8 mol m^−2^ day^−1^ (Figure 4c), as all plants were transitioned to identical greenhouse conditions with the same supplemental lighting. This uniformity in light environment during flowering indicates that any differences in final flower production and secondary metabolism were primarily driven by the disparity in light exposure during the vegetative period, rather than differences during reproductive development.

### 3.5. Yield, Electricity Use, and Economic Outcomes

Floral and total biomass yields reflected the morphological differences observed earlier in the study. Control plants produced 1295 g of flower compared to 1015 g in NB plants, representing a 21.6 percent advantage for the control treatment (Table 1). Stem and leaf biomass were also greater in the control group, resulting in a total post-flowering biomass of 2951 g compared to 2406 g in the NB group. These results indicate that the extended vegetative photoperiod in the control treatment produced more robust plants capable of supporting greater total biomass and floral yield.

Electricity consumption reflected the photoperiod treatments during the vegetative stage: the control used 540 kWh versus 390 kWh with NB, a 27.8% reduction in energy use (Table 2). At the 2024 average U.S. residential electricity rate of $0.1641/kWh, this reduction equates to an estimated cost savings of approximately $24.62 per growth cycle.

When yield and cost are considered together, the energy savings from NB treatment did not compensate for the revenue loss caused by reduced biomass. Using an average flower market value of $8.02 per gram, the yield difference between treatments represents a potential revenue gap of $2246.29 in favor of the control group. These findings highlight the trade-off between energy efficiency and production output in high value cannabis cultivation, suggesting that under the tested conditions and price assumptions night break is not economically justified for flower production.

## 4. Discussion

The primary objective of this experiment was to determine if the reduced DLI in the experimental NB group during the vegetative stage correlated with changes in plant biomass and flower production, as well as cannabinoid yield and concentration.

### 4.1. Light Intensity, Spectrum, and Photoperiod Considerations

In this study plants were grown under a mean PPFD of approximately 450 μmol m^−2^ s^−1^. While this level supported basic vegetative development, the previous literature indicates that *C. sativa* exhibits significantly higher rates of photosynthesis and biomass accumulation under elevated light intensities (up to 1500 μmol m^−2^ s^−1^) [17,22,25]. As such, our results likely underestimate the full production potential of NB treated plants under more optimized lighting. Future studies should investigate the interaction between night break photoperiods and high PPFD conditions, as increased irradiance may amplify photosynthetic activity and cannabinoid biosynthesis, particularly when paired with optimized DLI. Additionally, greenhouse cultivation with access to natural sunlight could provide the higher light intensities necessary to more accurately evaluate the agronomic potential of night break strategies under near-commercial conditions.

### 4.2. Night Break and Alternative Lighting Approaches

The treatment contrast arose from lowering the daily light integral (DLI) during vegetative growth while holding spectrum and instantaneous PPFD constant. The 1 h night break maintained a non-inductive state but reduced the daily photon dose, limiting carbon assimilation and aligning with the observed shorter height, internodes, branching, and lower floral mass. Combining the night break method with other lighting strategies may impact final yield. DE at very low PPFD is another method which can hold plants in the vegetative stage with minimal added DLI. In ‘Suver Haze’, extending a 9 h day to 15 h with 1–10 μmol m^−2^ s^−1^ suppressed full flowering but produced incomplete inflorescences, whereas ≥18 h at ~1 μmol m^−2^ s^−1^ prevented flowering [26]. Alternatives such as dimming lowers PPFD at a fixed photoperiod. At constant DLI, delivering the same photons over a longer photoperiod at lower PPFD increased Photosystem II (PSII) photochemical work (higher daily photosynthesis integral) compared with short, high PPFD days in lettuce, indicating longer-day delivery can be more efficient than shortening the day [27]. Adding inter-/sub-canopy lighting increases photon delivery to shaded tissues without changing daylength; in indoor medicinal cannabis, inter-canopy lighting increased dry inflorescence by ~30% and also boosted cannabinoid (THC) and total terpene yields while improving product uniformity, indicating that redistributing photons within dense canopies can raise effective DLI and chemical output [28].

### 4.3. Economic Implications of the Night Break Method

Economically, the control group’s higher flower yield comes at a higher electricity cost. Using the U.S. Energy Information Administration’s 2024 average residential electricity rate [29], the control group incurred an electricity cost of $88.61, while the experimental NB group’s cost was lower at $63.99. This results in a savings of $24.62 for the experimental group, representing a 27.8% reduction in electricity costs. While this study utilized a CBD producing hemp cultivar (Wife), it is worthwhile to consider the potential economic implications of a THC dominant cultivar grown under identical conditions. Using the most recent available data from the Connecticut General Assembly [30], the average price per gram of cannabis flower in four Northeast states (Connecticut ($10.62), Maine ($7.09), Massachusetts ($4.44), and New Jersey ($9.93)) was calculated to be $8.02.

Based on the average market value of cannabis flower ($8.02 per gram), the control group’s produced biomass (1295 g) would equate to approximately $10,386.50, while the NB group’s 1015 g yield would correspond to $8140.21, a difference of $2246.29 favoring the control. Although the NB treatment reduced electricity usage by 27.8 percent and saved $24.62 in energy costs, this modest savings does not offset the economic loss from reduced floral yield. The control group produced 21.6 percent more flower, a difference substantial enough to cover the added electricity cost and still return a higher profit. While the NB approach effectively prevented premature flowering and extended the vegetative phase, the reduced light exposure clearly correlated with lower total biomass, making the approach economically unviable in this context because the revenue loss from reduced yield was about ninety fold greater than the electricity savings.

## 5. Conclusions

These results demonstrate a clear trade-off between energy efficiency and plant performance. The higher biomass in the control group is attributable to longer light exposure, which supports photosynthesis and growth. The NB group’s lower yield was roughly proportional to the reduction in light received. While flower production was reduced, the NB group still achieved meaningful output, suggesting this method may be a practical option for growers prioritizing sustainability or operating under energy constraints. This balance between reduced input and modest output underscores the potential for integrating NB strategies in energy-conscious cannabis cultivation.

Although the NB treatment maintained functional vegetative growth, flower biomass was reduced compared to the control group. This decrease in yield outweighed the modest electricity savings, particularly when assessed using THC-market pricing benchmarks. These findings suggest that while the NB method may reduce operational costs, it does so at the expense of economic return in high-value production systems. The utility of the NB strategy may be more relevant in greenhouse environments, where natural sunlight can supplement reduced photoperiod lighting and partially offset declines in DLI. Future work should examine the interaction between NB treatments and increased light intensity, cultivar specific responses, and varying environmental conditions to assess the broader applicability of this approach. Studies under higher PPFD and mixed light conditions may clarify whether yield losses can be mitigated without sacrificing energy efficiency.

In summary, while the NB method offers clear energy savings, the associated reduction in yield limits its practicality for commercial cannabis cultivation where biomass production and cannabinoid output are primary economic drivers. These findings underscore the importance of evaluating input reduction strategies not only for sustainability, but also for their agronomic and economic viability in modern *C. sativa* production systems.

## Figures and Tables

**Table 1:** Total final harvest tissue weights by treatment. Values represent the total post-flowering biomass weights for each tissue type, measured at final harvest. Flower, stem, and total biomass were significantly greater in the control. Asterisks denote Control vs. NB significance from a two tailed paired *t* test: * *p* < 0.05.

Treatment	Flower Weight (g)	Stem Weight (g)	Leaf Weight (g)	Total Biomass (g)
Control	1295 *	1087 *	568	2951 *
Experimental	1015	912	479	2406

**Table 2:** Total electricity consumption and cost by treatment. Electricity consumption and associated costs were calculated based on measured kilowatt-hour usage and the 2024 U.S. average residential electricity rate as reported by the U.S. Energy Information Administration.

Treatment	Electricity Usage (kWh)	Cost ($)
Control	540	88.61
Experimental	390	63.99

## Data Availability

The original contributions presented in this study are included in the article. Further inquiries can be directed to the corresponding author.

## Abbreviations

The following abbreviations are used in this manuscript:

ANOVA	analysis of variance
CBC	cannabichromene
CBD	cannabidiol
CBDA	cannabidiolic acid (carboxylated)
CBG	cannabigerol
CBGA	cannabigerolic acid (carboxylated)
*C. sativa*	*Cannabis sativa*
DE	daylength extension
Δ^9^ THC	delta-9-tetrahydrocannabinol
DLI	daily light integral
HPLC	high performance liquid chromatography
HPS	high pressure sodium
LED	light emitting diode
NB	night break
PAR	photosynthetically active radiation
PPFD	photosynthetic photon flux density
SD	short day
SEM	standard error of the mean
THCA	tetrahydrocannabinolic acid
